# A two-stage solution

**DOI:** 10.7554/eLife.72980

**Published:** 2021-09-17

**Authors:** Fabien Guegan, Luisa Figueiredo

**Affiliations:** 1 Instituto de Medicina Molecular João Lobo Antunes Lisboa Portugal

**Keywords:** trypanosoma, sleeping sickness, tsetse fly, transmission, life cycle, development, Other

## Abstract

The parasite that causes African sleeping sickness can be transmitted from mammals to tsetse flies in two stages of its lifecycle, rather than one as was previously thought.

**Related research article** Schuster S, Lisack J, Subota I, Zimmermann H, Reuter C, Mueller T, Morriswood B, Engstler M. 2021. Unexpected plasticity in the life cycle of *Trypanosoma brucei*. *eLife* **10**:e66028. doi: 10.7554/eLife.66028

Many life-threatening pathogens – such as parasites, bacteria and viruses – use insects to pass from one mammal to the next (Wilson et al., 2020). For example, tsetse flies transmit *Trypanosoma brucei*, the parasite that causes the deadly disease known as African sleeping sickness. When the tsetse fly bites and sucks up the blood of an infected animal, it takes up the parasite which then undergoes various changes before getting injected into a new mammalian host when the insect feeds again.

*T. brucei* has a complex lifecycle involving multiple stages that allow the parasite to adapt to life within each host, and prepare for transmission into the next (Koch, 1909; Vickerman, 1985). In the mammalian host, *T. brucei* parasites initially travel to the bloodstream where they develop into a slender form that divides multiples times and disseminates to other tissues and organs (Krüger et al., 2018). As the population of parasites in the blood increases, density signals as well as other stimuli cause some of them to transition into a new, non-dividing form known as stumpy (Rojas et al., 2019; Vassella et al., 1997; Quintana et al., 2021; Zimmermann et al., 2017).

Both forms of the parasite are consumed when the tsetse fly takes blood from the mammalian host. The ingested *T. brucei* parasites then enter the midgut where they continue their lifecycle as they migrate back through the proventriculus (a valve-like organ that guards the entrance to the midgut) to then reach the salivary glands (Figure 1A).

**Figure 1.**
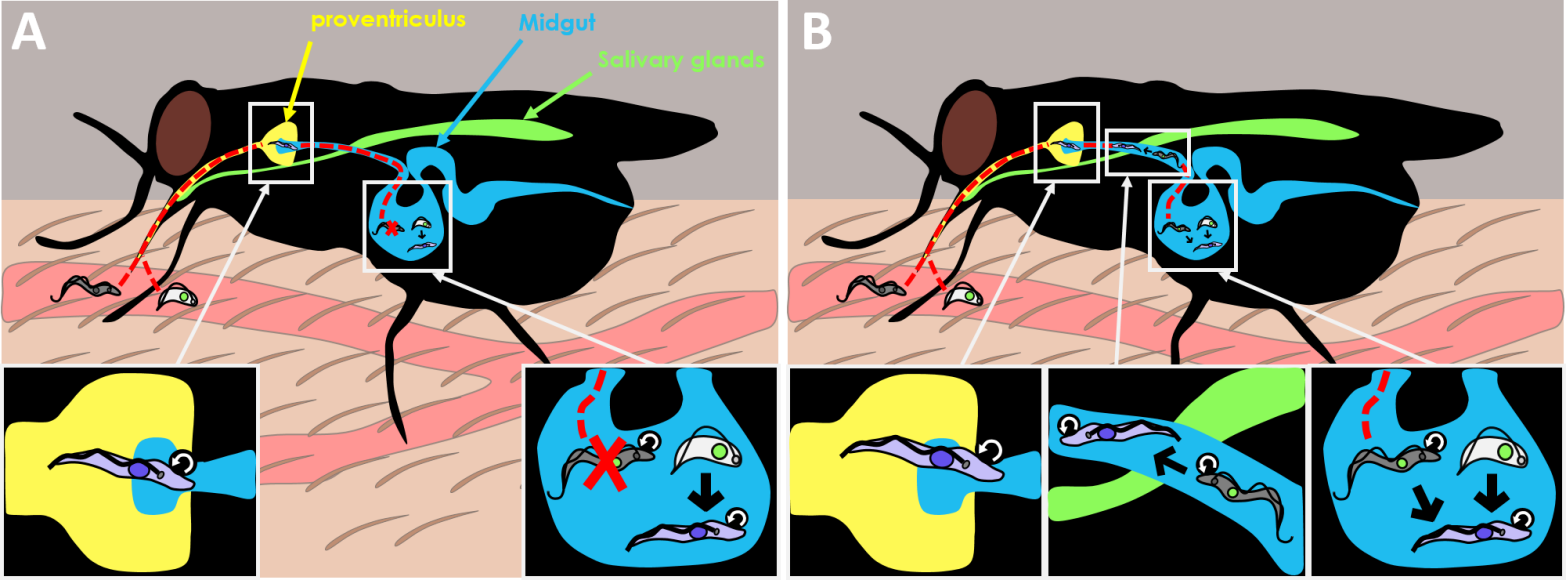
Old dogma and new theory in *T. brucei* transmission. (**A**) When the tsetse fly takes up blood from the mammalian host, it consumes two forms of the *Trypanosoma brucei* parasite: the slender form (gray) which is highly proliferative (represented by circular arrow), and the stumpy form (white) which is non-dividing. The old dogma states that only stumpy forms are able to survive and differentiate into the next stage of the life cycle, the procyclic form (purple), as slender forms cannot survive the harsh conditions of the midgut (bottom right inset). The procyclic form then enters the proventriculus (yellow; bottom left inset) and migrates to the salivary glands (green) where it develops into the more infectious metacyclic form which the fly injects into a new mammal when it feeds again. (**B**) The new theory put forward by Schuster et al. shows that slender forms of the parasite are also able to survive and develop inside the tsetse fly. Slender forms were found to differentiate directly into the procyclic stage (bottom right inset), which probably improves the success of the tsetse infection. Schuster et al. also found that the highly motile slender form can reach the entry of the proventriculus, and they hypothesize that this migration happens as the parasite transitions to the next stage of the lifecycle (bottom middle inset).

Image credit: Fabien Guegan.

Previous research has shown that parasites in the stumpy form are resistant to the digestive effects of proteases in the midgut and are better equipped to use the energy sources available in the fly (Silvester et al., 2017). Furthermore, parasites usually enter the non-dividing stumpy stage before differentiating into the next phase of the lifecycle, the procyclic form. These findings have led to the belief that only parasites in the stumpy form are able to infect the tsetse fly and progress through the lifecycle, establishing a scientific dogma about parasite transmission.

However, infected mammals typically only have small amounts of parasites in their blood, as *T. brucei* will have spread to other tissues and organs. Therefore, the chances of a tsetse fly ingesting parasites during a blood meal is very low, particularly those in the stumpy form, which can only survive in the blood for three days before dying (Turner et al., 1995). Now, in eLife, Markus Engstler and colleagues from the University of Würzburg – including Sarah Schuster, Jaime Lisack and Ines Subota as joint first authors – report new findings that help explain how African sleeping sickness is able to persist when transmission appears so unlikely (Schuster et al., 2021).

The team were originally interested in comparing the transmission efficiency of stumpy forms that had been generated by different stimuli, using the slender stage as a negative control for their experiments. The insects were fed with blood containing parasites in the slender or stumpy form which can be distinguished by a fluorescent marker that is only expressed in the stumpy stage. Unexpectedly, Schuster et al. found that parasites in the slender form were able to survive the harsh conditions of the insect midgut and went on to infect the proventriculus and the salivary glands.

Next, Schuster et al. set out to determine how many parasites of each form are required for infection by limiting the number of *T. brucei* parasites present in the blood fed to the flies. The experiments showed that only a single slender or stumpy form is needed for the parasite to be sufficiently transmitted between mammals and flies. These findings not only challenge the predominant theory that *T. brucei* needs to be in the stumpy form to successfully infect tsetse flies, but they also show how transmission can take place when the concentration of parasites in the blood is incredibly low.

Finally, Schuster et al. found that when slender forms reach the tsetse midgut, they can directly differentiate into the procyclic form without having to pass through the stumpy stage (Figure 1B). This result contradicts the textbook lifecycle of *T. brucei*, in which slender forms have to transition to the cell-cycle arrested stumpy stage before entering the next phase of the lifecycle. Thus, this work raises important questions about the role of the stumpy form in parasite biology.

These findings help solve the long-standing paradox of how mammals with low numbers of *T. brucei* parasites in their blood can still infect tsetse flies. In the future, it will be necessary to test if this behavior is also present in other subspecies of *T. brucei*, especially those which cause disease in humans, such as *T. brucei gambiense* and *T. brucei rhodesiense*. Finding more genes that are specifically activated in slender, stumpy and other forms of the parasite would also help researchers distinguish between the different stages of the lifecycle. These additional molecular markers would make it easier to follow the temporal changes that occur in vivo when parasites colonize the tsetse fly.
